# Infections and antimicrobial prescribing in patients hospitalized with coronavirus disease 2019 (COVID-19) during the first pandemic wave

**DOI:** 10.1017/ash.2023.135

**Published:** 2023-04-17

**Authors:** Lynn Chan, Simran Gupta, Alicia J. Sacco, Sabirah N. Kasule, Hally Chaffin, Fionna F. Feller, Lanyu Mi, Elisabeth S. Lim, Maria Teresa Seville

**Affiliations:** 1 Department of Pharmacy, Ronald Reagan UCLA Medical Center, Los Angeles, California; 2 Department of Internal Medicine, Mayo Clinic Hospital, Phoenix, Arizona; 3 Department of Pharmacy, Mayo Clinic Hospital, Phoenix, Arizona; 4 Department of Quantitative Health Sciences, Division of Clinical Trials and Biostatistics, Mayo Clinic Arizona, Scottsdale, Arizona; 5 Division of Infectious Diseases, Yale New Haven Hospital, New Haven, Connecticut; 6 Division of Infectious Diseases, Mayo Clinic Hospital, Phoenix, Arizona

## Abstract

**Objective:**

To evaluate the rate of coinfections and secondary infections seen in hospitalized patients with COVID-19 and antimicrobial prescribing patterns.

**Methods:**

This single-center, retrospective study included all patients aged ≥18 years admitted with COVID-19 for at least 24 hours to a 280-bed, academic, tertiary-care hospital between March 1, 2020, and August 31, 2020. Coinfections, secondary infections, and antimicrobials prescribed for these patients were collected.

**Results:**

In total, 331 patients with a confirmed diagnosis of COVID-19 were evaluated. No additional cases were identified in 281 (84.9%) patients, whereas 50 (15.1%) had at least 1 infection. In total, of 50 patients (15.1%) who were diagnosed with coinfection or secondary infection had bacteremia, pneumonia, and/or urinary tract infections. Patients who had positive cultures, who were admitted to the ICU, who required supplemental oxygen, or who were transferred from another hospital for higher level of care were more likely to have infections. The most commonly used antimicrobials were azithromycin (75.2%) and ceftriaxone (64.9%). Antimicrobials were prescribed appropriately for 55% of patients.

**Conclusions:**

Coinfection and secondary infections are common in patients who are critically ill with COVID-19 at hospital admission. Clinicians should consider starting antimicrobial therapy in critically ill patients while limiting antimicrobial use in patients who are not critically ill.

The coronavirus disease 2019 (COVID-19) pandemic, caused by severe acute respiratory syndrome coronavirus 2 (SARS-CoV-2), challenged healthcare systems to adapt to evolving infection prevention and control and therapeutic recommendations and diverted antimicrobial stewardship efforts to the pandemic response. At the start of the pandemic, healthcare providers were prescribing antibiotics based on evidence (1) that 18%–30% of bacterial coinfections co-occur with viral respiratory infections such as severe influenza,^1–4^ (2) that morbidity and mortality is high in patients with bacterial coinfections with severe influenza, and (3) that differentiating SARS-CoV-2 infection from bacterial pneumonia is challenging because patients often present with similar symptoms and abnormalities on chest imaging.^5^

As the pandemic continued, studies showed a low prevalence of bacterial coinfection in patients with COVID-19. In a meta-analysis of 24 studies by Langford et al,^6^ ∼3.5% of patients with COVID-19 had bacterial coinfection at presentation and 14.3% of patients developed a secondary bacterial infection.^6^ In a review of 18 studies by Rawson et al,^7^ only 8% of patients had bacterial coinfections at hospital admission; however, 72% received antimicrobial therapy.

Also, critically ill patients with COVID-19 are susceptible to the development of secondary bacterial and fungal infections due to prolonged hospitalization, presence of invasive medical devices, and drug-induced immunosuppression. In a multicenter study by Rouze et al,^8^ the incidence of ventilator-associated pneumonia (VAP) in patients with COVID-19 was 50%.^8^ In another multicenter study by Russell et al,^9^ 70.6% of patients with COVID-19 had secondary infections and the antimicrobial prescribing rate was 85.2% during the study period.^9^

The disproportionally high rate of antimicrobial prescribing in the setting of a low prevalence of bacterial coinfection places a high burden on antimicrobial stewardship programs, places patients at avoidable risk of toxicity from antibiotics, and can lead to antimicrobial resistance.^10^

In this study, we characterized the rate of coinfections and secondary infections in hospitalized patients with COVID-19, with a focus on clinical outcomes and antimicrobial utilization. We sought to determine appropriate or inappropriate use of antimicrobial therapy.

## Methods

This single-center, retrospective cohort study included all patients aged ≥18 years admitted between March 1, 2020, and August 31, 2020, to a 280-bed, academic, tertiary-care hospital. All patients hospitalized from late March 2020 onward had nasopharyngeal swabs for SARS CoV-2 polymerase chain reaction (PCR) testing on admission and all those who tested positive and were hospitalized for at least 24 hours were included.

Data obtained from the medical record included demographics, comorbidities, oxygenation status, chest imaging, indwelling medical devices, microbiology, laboratory results at admission, medications including COVID-19 therapy immunosuppressants, and antimicrobials within 30 days of admission and during the hospitalization, mortality, and readmission within 30 days after hospital discharge.

Microbiology results from blood, respiratory, and urinary specimens were included. Antimicrobial susceptibility testing was performed with BD Phoenix automated identification and susceptibility testing system (Becton-Dickinson, Franklin Lakes, NJ).

Patients with organisms identified on microbiologic testing of blood, respiratory, and urine, specimens were reviewed for the presence of infection using the 2020 National Healthcare Safety Network (NHSN) Patient Safety Component Manual definitions of bacteremia, pneumonia, and urinary tract infections.^11^ Coinfection was defined as infection onset before hospital day 3 and secondary infection was defined as infection with onset on hospital day 3 or later. Coinfections and secondary infections were aggregated in the analysis because of the small number of infections.

Antimicrobial use was deemed inappropriate when antibiotics were used for colonization or contaminated cultures, when there was a lack of de-escalation following susceptibility results, or if an antimicrobial prescribed was not effective for the isolated pathogen.

The study was approved by the Mayo Clinic Institutional Review Board.

### Statistical analysis

Patient characteristics were summarized as median with interquartile range for continuous variables or count with percentage for categorical variables. Comparisons were made between groups using Wilcoxon rank-sum test or the Fisher exact test as appropriate. Multivariable logistic regression was used to investigate the association between the outcome of infection identification and patient clinical characteristics factors. The analysis was conducted using RStudio version 4.0.3 software (RStudio Team, PBC, Boston, MA, 2022). All tests were 2-sided and *P* values <.05 were considered significant.

## Results

**Fig. 1.**
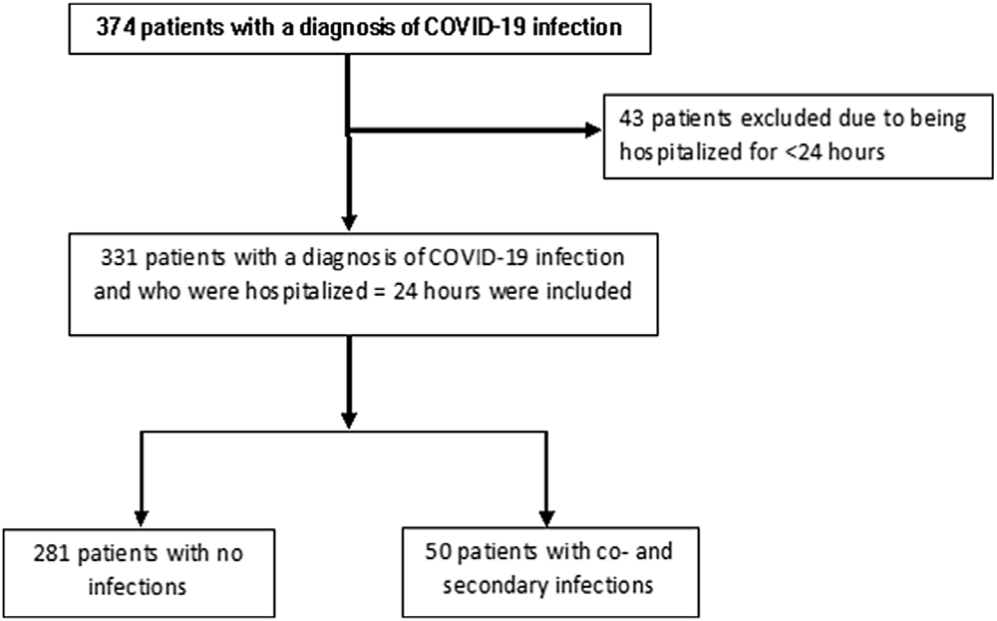
Patient selection.

In total, 331 patients with confirmed diagnosis of COVID-19 were evaluated (Fig. 1). The median patient age was 60.0 (IQR, 48.0–72.0) years and 202 (61.0%) patients were male. Moreover, 243 patients (73.6%) were admitted to the hospital from the emergency department, and 80 (24.2%) were transferred from outside hospitals (OSH) for higher level of care (*P* < .001) (Table 1). There were no significant differences in comorbidities such as diabetes mellitus, immunodeficiency, transplantation or underlying structural lung disease between patients with or without infection. Patients who were on steroids for any reason prior to admission were more likely to have an infection (36% vs 14.9%; *P* = .001).

**Table 1.** Baseline Characteristics of Patients With or Without Infection

Characteristic	No Infection (N=281)	Infection (N=50)	Total (N=331)	*P* Value
Age, median y (IQR)	60.0 (48.0–71.0)	63.0 (48.8–76.0)	60.0 (48.0–72.0)	.44
Sex, male, no. (%)	171 (60.9)	31 (62)	202 (61.0)	>.99
**Ethnicity, no. (%)**				.23
White	156 (55.9)	23 (46.9)	179 (54.6)	
Hispanic or Latino	56 (20.1)	8 (16.3)	64 (19.5)	
American Indian or Alaska Native	45 (16.1)	14 (28.6)	59 (18.0)	
Other	22 (7.9)	4 (8.2)	26 (7.9)	
BMI, median (IQR)	29.4 (25.7–33.8)	29.0 (24.7–33.2)	29.4 (25.5–33.7)	.42
**Comorbidities, no. (%)**				
Diabetes mellitus	86 (31.4)	21 (42.9%)	107 (33.1)	.14
Cancer on active chemotherapy	16 (5.9)	2 (4.3)	18 (5.6)	>.99
Chronic kidney disease	49 (17.9)	8 (16.7)	57 (17.7)	>.99
Cardiovascular disease	59 (21.4)	14 (28.6)	73 (22.5)	.27
Hypertension	136 (48.6)	23 (47.9)	159 (48.5)	>.99
Structural lung disease	20 (7.3)	2 (4.3)	22 (6.9)	.75
**Transplantation, no. (%)**				
Hematological stem-cell transplant	6 (2.2)	0 (0.0)	6 (1.9)	.60
Solid-organ transplant	32 (11.7)	7 (14.6)	39 (12.1)	.63
**Immunosuppressant agents, no. (%)**				
Steroids	42 (14.9)	18 (36.0)	60 (18.1)	.001
Other immunosuppressants^a^	38 (13.5)	11 (22.0)	49 (14.8)	.13
**Labs at admission, median (IQR)**				
WBC	6.3 (4.6–8.5)	7.8 (5.1–11.2)	6.5 (4.6–8.8)	.003
Lymphocytes	0.9 (0.6–1.2)	0.9 (0.5–1.2)	0.9 (0.6–1.2)	.55
CRP	68.2 (30.1–124.9)	98.8 (50.4–160.0)	71.5 (30.3–128.0)	.03
Ferritin	591.0 (254.5–1,043.5)	709.0 (303.0–1,003.5)	608.5 (258.0–1,038.2)	.71
Lactate	1.4 (1.1–1.7)	1.6 (1.1–1.9)	1.4 (1.1–1.8)	.20
**Admission status, no. (%)**				<.001
ICU	18 (6.4)	17 (34.0)	35 (10.6)	
Progressive care unit	80 (28.6)	13 (26.0)	93 (28.2)	
Medical floor	182 (65.0)	20 (40.0)	202 (61.2)	
**Location admitted from, no. (%)**				<.001
Direct admit	5 (1.8)	2 (4.0)	7 (2.1)	
ED	219 (78.2)	24 (48.0)	243 (73.6)	
Transfer from outside hospital	56 (20.0)	24 (48.0)	80 (24.2)	
**Oxygenation at admission, no. (%)**				<.001
HFNC	20 (7.2)	5 (10.0)	25 (7.6)	
LFNC	143 (51.3)	18 (36.0)	161 (48.9)	
Ventilator	6 (2.2)	14 (28.0)	20 (6.1)	
None	110 (39.4)	13 (26.0)	123 (37.4)	
ECMO, no. (%)	3 (6.2)	9 (39.1)	12 (16.9)	.001
Presence of consolidation or groundglass opacities on chest imaging, no. (%)	220 (78.9)	46 (92.0)	266 (80.9)	.03
**Invasive medical devices, no. (%)**				
Urinary catheters	66 (23.5)	27 (54.0)	93 (28.1)	<.001
Endotracheal tube	20 (7.1)	23 (46.0)	43 (13.0)	<.001
Central venous catheters	41 (14.6)	25 (50.0)	66 (19.9)	<.001

Note. CRP, C-reactive protein; ECMO, extracorporeal membrane oxygenation; ED, emergency department; HFNC, high-flow nasal cannula; ICU, intensive care unit; IQR, interquartile range; LFNC, low-flow nasal cannula; WBC, white blood cells.

a Other immunosuppressant agents include: abatacept, adalimumab, anakinra, azathioprine, certolizumab, IV/PO chemotherapy, cyclosporine, etanercept, everolimus, infliximab, leflunomide, mycophenolate, natalizumab, rituximab, sirolimus, tacrolimus, tofacitinib, vedolizumab

Of the 331 patients, 281 (84.9%) had no coinfection or secondary infection and 50 (15.1%) had at least 1 infection. Of the 50 patients with infections, 17 (34.0%) were admitted to the ICU: 13 (26.0%) in the progressive care unit and 20 (40.0%) on the medical floor. Also, 25 patients (7.6%) had coinfections, 24 patients (7.3%) had secondary infections, and 1 patient (0.3%) had both a coinfection and a secondary infection. Patients who were admitted to the ICU were more likely to have an infection (*P* < .001). Of the 281 patients with no infection, 65% were admitted to the medical floor and 6.4% were admitted to the ICU. The median ICU length of stay was significantly longer for patients with infections at 28.0 days (IQR, 16.0–51.0) compared to patients without infection at 6.0 days (IQR, 2.5–12.0; *P* < .001).

On admission, compared to patients with no infection, patients with infections had a greater requirement for supplemental oxygenation (74.0% with infection vs 60% without infection) and abnormal chest imaging (92.0% with infection vs 78.9% without infection). These patients also had more invasive medical devices: urinary catheters (54.0% with infection vs 23.5% without infection), central venous catheters (50.0% with infection vs 14.6% without infection), and endotracheal tubes (46.0% with infection vs 7.1% without infection; *P* < .001). Length of hospital stay was longer in patients with infections compared to those without: 20.5 days (IQR, 6.0–35.0) versus 6.0 days (IQR, 3.0–10.0 days; *P* < .001) (Table 2). Patients who had infections were more likely to be readmitted to the hospital within 30 days of discharge (20.0% vs 9.3%; *P* = .05).

**Table 2.** Outcomes of Patients With or Without Infection

Outcome	No Infection (n=281)	Infection (n=50)	Total (n=331)	*P* Value
Length of hospitalization, median d (IQR)	6.0 (3.0–10.0)	20.5 (6.0–35.0)	6.0 (4.0–11.5)	<.001
Length of ICU stay, median d (IQR)	6.0 (2.5–12.0)	28.0 (16.0–51.0)	10.0 (3.8–27.2)	<.001
COVID-19 Therapies, no. (%)^a^	200 (71.9)	38 (76.0)	238 (72.6)	.61
Readmission within 30 d of discharge, no. (%)	26 (9.3)	10 (20.0)	36 (10.9)	.05
Death during hospitalization, no. (%)	24 (8.6)	9 (18.0)	33 (10.0)	.07

Note. IQR, interquartile range; ICU, intensive care unit.

a COVID-19 therapies include convalescent plasma, dexamethasone, hydroxychloroquine, lenzilumab, remdesivir, and tocilizumab.

**Fig. 2.**
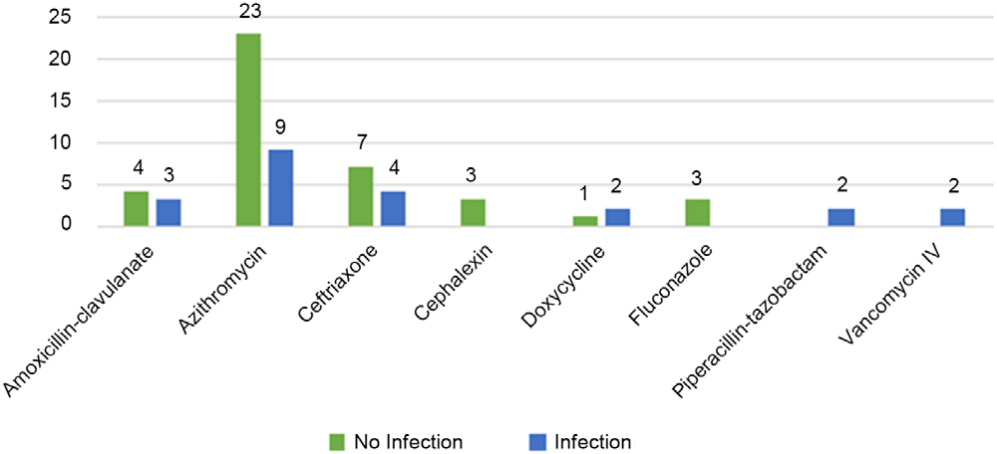
Most frequently prescribed antimicrobials within 30 days prior to admission.

Moreover, 56 patients (17.0%) received antibiotics within 30 days prior to admission for COVID-19 and of those, 16 (32.7%) patients had infection during hospitalization. The most commonly prescribed antibiotics before and during hospitalization were azithromycin and ceftriaxone (Fig. 2).

**Fig. 3.**
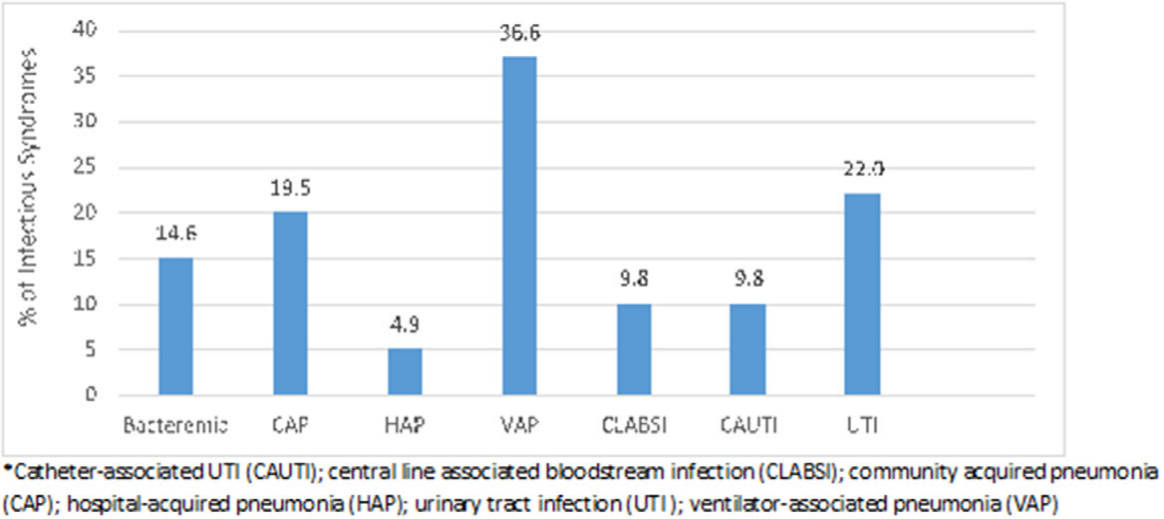
Infectious disease syndromes identified. Note. CAUTI, catheter-associated UTI; CLABSI, central-line–associated bloodstream infection; CAP, community-acquired pneumonia; HAP, hospital-acquired pneumonia; UTI, urinary tract infection; VAP, ventilator-associated pneumonia.

The most common types of infection were ventilator-associated pneumonia (36.6%), followed by urinary tract infection (22.0%), community-acquired pneumonia (19.5%), and bacteremia (14.6%) (Fig. 3).

**Fig. 4.**
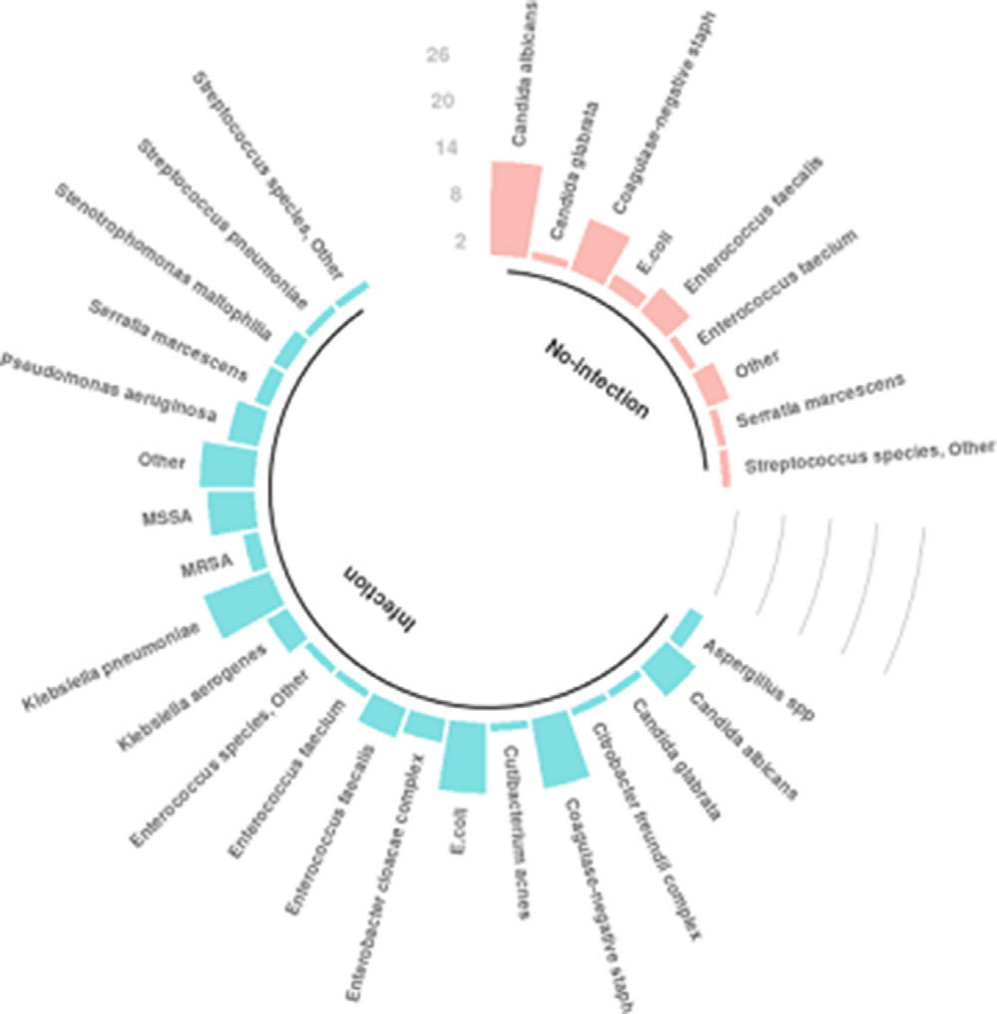
Organisms identified from cultures.

In total, 66 patients (19.9%) had positive cultures. Among them, 25 patients (8.9%) without an infection had at least 1 positive culture, and 41 patients (82%) with an infection had at least 1 positive culture along with criteria for infection (*P* < .001). The most common organisms identified in patients who did not have infection were *Candida albicans*, coagulase-negative *Staphylococci* (CoNS) and *Enterococcus faecalis* whereas *Klebsiella pneumoniae*, *Escherichia coli*, *Staphylococcus aureus* (methicillin resistant and methicillin susceptible), and CoNS were the most common organisms identified in patients who had infections (Fig. 4).

The prevalence of antibiotic use was 100% in those with infections and 68.3% in patients without infections. Patients with coinfections or secondary infections were treated with a median of 5.0 antimicrobials compared to 2.0 in patients without infection (*P* < .001). The most commonly used antimicrobials were azithromycin (75.2%) and ceftriaxone (64.9%) in patients with or without infection. Vancomycin, cefepime, and meropenem were administered to 60.0%, 42.0% and 34.0% of patients, respectively, who had coinfection or secondary infection (Table 3).

**Table 3.** Commonly Used Antimicrobial Agents During Hospitalization

Antimicrobial	No Infection (N=281), No. (%)	Infection (N=50), No. (%)	Total (N=331), No. (%)	*P* Value
Azithromycin	146 (76.0)	36 (72.0)	182 (75.2)	.58
Caspofungin	4 (2.1)	6 (12.0)	10 (4.1)	.006
Cefazolin	1 (0.5)	6 (12.0)	7 (2.9)	<.001
Ceftriaxone	132 (68.8)	25 (50.0)	157 (64.9)	.02
Cefepime	23 (12.0)	21 (42.0)	44 (18.2)	<.001
Ertapenem	0 (0.0)	7 (14.0)	7 (2.9)	<.001
Fluconazole	12 (6.2)	11 (22.0)	23 (9.5)	.002
Meropenem	13 (6.8)	17 (34.0)	30 (12.4)	<.001
TMP/SMX	3 (1.6)	5 (10.0)	8 (3.3)	.01
Vancomycin IV	47 (24.5)	30 (60.0)	77 (31.8)	<.001

Note. TMP/SMX, trimethoprim-sulfamethoxazole; IV, intravenous.

Overall, 154 positive cultures were reviewed to determine the appropriateness of antimicrobial use. Among these cultures, 100 were from patients with infections and 54 were from patients without infection. Antimicrobials were prescribed inappropriately for the pathogen identified in 45 patients (45%) with infections, and the most common reasons were lack of de-escalation, treatment of colonization, and contaminated cultures.

## Discussion

In this study, most patients had no infection, but we identified coinfections and secondary bacterial infections in 50 hospitalized patients (15.1%) with COVID-19 during the first wave of the COVID-19 pandemic. Of the 50 COVID-19 patients identified with infections, 25 (7.6%) had coinfection and 24 (7.3%) had secondary infection; 1 patient (0.3%) was identified as having both coinfection and secondary infections. These results are similar to reported rates from 6% to 29%.^6,10,12^ Similarly, Westblade et al^13^ reported that <4% of patients with COVID-19 had documented bacterial coinfections on hospital admission.^13^ Ripa et al^10^ found an overall 28-day cumulative incidence of secondary infections of ∼16.4%, with more bloodstream infections (7.9%) than respiratory tract infections (3.0%).^10^

Severe hypoxemia, severe lymphopenia, need for intensive care in the first 48 hours after hospital admission, and receipt of steroids have been reported to be predictive factors for secondary infections in patients with COVID-19.^10,14^ In our study, COVID-19 patients with the following factors were more likely to have infections: those who were transferred from OSH for higher level of care, those who required supplemental oxygenation with HFNC or mechanical ventilation at admission, those who had invasive medical devices, those who had consolidation or ground-glass opacities on initial imaging, and those who had longer lengths of hospitalization.

We did not find a difference in mortality during hospitalization in patients with COVID-19 with or without infection. This may be because of thorough investigations and effective therapy provided during hospitalization. However, we did find a higher 30-day readmission in patients who had infections, which could be related to patient comorbidities.

Also 17.0% of patients had antimicrobial exposure within 30 days prior to hospitalization, and azithromycin was the most prescribed agent. Of the patients who had antimicrobial exposure 30 days prior to admission, 28.6% had infection during hospitalization for COVID-19. Patients hospitalized with COVID-19 who had no coinfections were started on empiric azithromycin (76.0%) and ceftriaxone (68.8%). This finding is similar to those reported by Vaughn et al,^15^ which showed that 56.6% were treated with early empiric antibacterial therapy despite only finding 3.5% of patients with community-onset bacterial coinfection. Routine use of empiric antibiotics for COVID-19 patients has since been discouraged on institutional protocols, with improvement in antimicrobial prescribing.

Broad-spectrum antimicrobials were utilized commonly in patients with infections. In our study, 45% of antimicrobials were inappropriately prescribed. Guidelines recommend broad-spectrum empiric antimicrobials in critically ill patients; however, antimicrobial therapy should be re-evaluated as microbiology results become available. With the ongoing pandemic, it will be necessary for antimicrobial stewardship programs to monitor the utilization of antimicrobial agents to ensure appropriate antimicrobial use in patients hospitalized with COVID-19.

Our study had several limitations. This study was conducted retrospectively, and the results are subject to potential sources of bias and confounding inherent to retrospective studies. This study was single-center retrospective study conducted at a tertiary-care hospital during the first wave of the COVID-19 pandemic. These findings may not be generalizable because of variations in microbiological epidemiology and because management strategies have evolved. Only patients who had positive cultures to determine coinfection and secondary infections were evaluated, which may have underrepresented the true infection rates. Timing of initiation and duration of antimicrobials, the frequency of antimicrobial changes during treatment and rates of resistance were not evaluated in this study. Finally, we utilized CDC NHSN surveillance definitions, which may not reflect clinical practice.

In conclusion, whereas most of our patients did not have any infection, coinfections and secondary infections were diagnosed in 15.1% of hospitalized patients with COVID-19. COVID-19 patients with the following factors had more infections: those who needed ICU admission, those who required supplemental oxygen, those who had consolidation or ground-glass opacities on imaging, underwent prolonged hospitalization, and those who had invasive medical devices. Initiating empiric antimicrobials may be reasonable for these patients. Further study of infection and antimicrobial use in patients hospitalized for COVID-19 could help inform appropriate antimicrobial stewardship efforts in these patients.
